# Healing the heart, one variant at a time

**DOI:** 10.1016/j.omtn.2024.102407

**Published:** 2024-12-07

**Authors:** Sadia Mohsin, Xiaofeng Yang, Hong Wang, Mohsin Khan

**Affiliations:** 1 Aging and Cardiovascular Discovery Center, Lewis Katz School of Medicine, Temple University, Philadelphia, PA 19140, USA; 2 Lemole Cener for Integrated Lymphatics and Vascular Research, Lewis Katz School of Medicine, Temple University, Philadelphia, PA 19140, USA; 3 Center for Metabolic Disease Research, Lewis Katz School of Medicine, Temple University, Philadelphia, PA 19140, USA; 4 Department of Cardiovascular Sciences, Lewis Katz School of Medicine, Temple University, Philadelphia, PA 19140, USA

## Main text

The limited capacity of the heart to replace dead cardiomyocytes following injury has driven researchers to explore novel approaches that promote cardiac repair and regeneration. Extracellular vesicles (EVs) have emerged as a viable option for cardiac regenerative medicine^1^ and carry equal if not more potency compared to cell type of origin. EVs are enriched in non-coding RNAs (ncRNAs), coding RNAs, and proteins. Several benefits have been identified for EV-based approaches that repair the injured myocardium, including low immunogenicity and an ability to deliver a diverse payload comprising small yet powerful signaling molecules such as non-coding RNAs. Nevertheless, EVs derived, for instance, from stem cells such as cardiosphere-derived cells are enriched with over 10,000 distinct ncRNAs^2^ with potentially overlapping or antagonistic functions. Moreover, there is limited information on the enrichment of long ncRNA (lncRNA) splice variants within EVs and their impact on cardiac biology. In this respect, Vilaca et al. provide evidence for loading of lncRNA splice variants in EVs derived from Wharton jelly mesenchymal stromal cells (WJ-MSCs) upon engineering parent cells with lncRNA and their specific targeting of cardiac cells upon delivery to the heart.

lncRNAs were first identified as long RNAs, with little or no protein coding potential during global transcriptomic analysis. lncRNAs are a group of heterogeneous RNAs more than 200 nt long and can regulate gene expression via binding to microRNAs, RNAs, and DNA.^3^ In the context of cardiac biology, lncRNAs are found in all cells of the heart such as cardiomyocytes, endothelial cells, vascular smooth muscle cells, and fibroblasts. During cardiac development, changes in the expression of lncRNAs such as UpperHand and *Fendrr* can result in embryonic lethality and cardiac structural defects.^4^ Several lncRNAs have been implicated in the pathogenesis of cardiovascular diseases (CVDs) that can impact hypertrophic growth in response to injury, impaired cardiac contractility, and increased remodeling in response to myocardial injury in animal models of CVD. These studies highlight the diverse role played by lncRNAs in cardiovascular development and response to injury. One of the widely studied lncRNAs in the heart is lncRNA-H19, known to regulate cardiomyocyte numbers and proliferation during cardiac development.^5^ In the failing heart, lncRNA-H19 is known to downregulate while its therapeutic overexpression attenuates adverse cardiac remodeling in a transaortic constriction model for cardiac injury.^6^ It has been shown that EV-based lncRNA-H19 therapy can regenerate injured myocardial tissue. Nevertheless, there is limited information as to whether lncRNA-H19 splice variants are enriched in EVs, including their physiological and pathological impact on the heart.

The study by Vilaca and colleagues^7^ identifies how EVs from WJ-MSCs are enriched with not only lncRNA-H19 but also different sets of H19 splice variants. lncRNAs are known to be alternatively spliced into splice variants that can interact with small peptides or can become circular RNAs. To show the physiological relevance of lncRNA-H19 splice variants in the heart, Vilaca et al. design specific primers for 16 splice variants of lncRNA-H19 and confirm their expression to be decreased in the heart during transition from embryonic stages to adult. Moreover, they found that the various lncRNA-H19 splice variants are differentially expressed in the heart, further highlighting their physiological relevance. It is known that EVs can be engineered to carry proteins, RNAs, and ncRNAs, including lncRNAs such as H19. However, there is limited information as to whether lncRNA splice variants are enriched within EVs upon engineering parent cells with lncRNAs. To address this limitation, Vilaca et al. transfected WJ-MSCs with mammalian expression vectors for lncRNA-H19. Interestingly, all lncRNA-H19 splice variants were expressed in EVs at approximately similar levels, even for variants that were less expressed in WJ-MSCs.

One of the main questions regarding any EV-based delivery strategy is what primary cell type is being targeted by the transplanted EVs in the heart. Vilaca et al. utilize WJ-MSCs because of their low immunogenicity, test EV uptake, and RNA transfer in various cardiac cells such as neonatal mouse cardiomyocytes, fibroblasts, and mouse aortic endothelial cells (MAECs). Fibroblasts showed significantly higher EV internalization than did cardiomyocytes and MAECs, but it is unclear whether higher uptake results in increased RNA transfer, as the authors note similar splice variant expression in all cardiac cell types. To test *in vivo* EV uptake and RNA transfer, Vilaca et al. utilize cardiac slices previously shown as a three-dimensional microphysiological system able to preserve cellular composition and mimic heart physiology *in vitro*. Cardiac slices were exposed to hypoxia/reoxygenation followed by administration of PKH-67-labeled EVs. Within 24 h of treatment, the majority of cardiac cells with EVs were cardiomyocytes and endothelial cells, with small amounts in other cells, possibly fibroblasts. These results seem to contrast earlier findings by the authors using cardiac cell types treated with EVs *in vitro*, possibly suggesting variability between cardiac slices and the cells lines employed.

EVs have emerged as efficient drug delivery vehicles, able to carry adeno-associated viruses, proteins, and RNAs to target cells and organs.^8^ Recent studies have engineered parent cells with lncRNAs to subsequently load EVs with them. Since alternative splicing of lncRNAs can generate splice variants that can affect signaling pathways, the Vilaca et al. study provides novel information showing how engineering parent cells with lncRNAs can lead to enrichment of splice variants in EVs in addition to the lncRNA itself. With identification of new lncRNAs in the heart, the field of cardiovascular lncRNA biology has advanced rapidly. As 70% of human lncRNAs do not have homologs in other species, it is important to consider the relevance of lncRNAs and associated signaling mechanisms in humans, including selection of cell and animal models that exhibit human translational value. Vilaca et al. note that heterogeneous nuclear ribonucleoprotein A2B1 promotes lncRNA-H19 sorting into EVs; however, this mechanism was not tested in the paper, and whether it can be applied to sorting of splice variants into EVs is unknown. It is well established that ncRNAs, including lncRNA, are not homogenously packed within EVs and the same could be true for lncRNA splice variants. Therefore, it is essential to employ the latest techniques that allow analysis of single EV heterogeneity.^9^ Vilaca et al. utilize PKH-67-labeled EVs for their analysis of uptake in cardiac cells using histological sections from cardiac slices. However, immunofluorescent signals from the PKH-67-labeled EVs in sections can overlap with autofluorescence in various tissues,^10^ complicating data interpretation. Future studies should use caution and consider alternatives for using PKH dyes for EV tracking in tissues. Results showing increased uptake in cardiac fibroblasts versus cardiomyocytes in the cardiac slices indicate potential disparity between the main cardiac cell types targeted by the WJ-MSC EVs.

Overall, the findings by Vilaca et al. provide new insights into the packaging of lncRNA-H19 splice variants into EVs upon engineering parent cells with lncRNA-H19 and the cardiac tropism of WJ-MSC-derived EVs for delivery of lncRNA-H19 to cardiac cells. These findings highlight the importance of EVs enriched with lncRNA splice variants that will encourage future studies on delineating the physiological and pathological impacts of lncRNA splice variants in the heart.

## Acknowledgments

This work was supported by American Heart Association Transformational Project Award 20TPA35490355 to M.K.

## Declaration of interests

The authors declare no competing interests.
